# Severe Hypothyroidism-Induced Volvulus

**DOI:** 10.14740/jocmr2357w

**Published:** 2015-10-23

**Authors:** Rafay Khan, Amar Ahmed, Sunil Tulpule, Kalyani Regeti, Shuvendu Sen, Teena Mathew

**Affiliations:** a Department of Internal Medicine, Raritan Bay Medical Center, 530 New Brunswick Ave, Perth Amboy, NJ, USA

**Keywords:** Hypothyroidism, Gastrointestinal, Motility, Volvulus, Thyroid

## Abstract

Thyroid disorders have been found to be associated with multiple organ systems and thus have a broad spectrum of presenting symptoms and clinical conditions. Certain aspects of the gastrointestinal (GI) system have yet to be fully understood and documented. Hypothyroidism and even hyperthyroidism have been identified in patients with motility symptoms involving the GI tract. These symptoms can vary and can be a complication of undertreated or undiagnosed condition involving the thyroid. Unfortunately, the mechanism in which these hormones can impact intestinal motility remains poorly understood and not well documented. In this case report, we discuss the presentation of a 71-year-old female with poorly managed hypothyroidism presenting with significant abdominal distention and pain secondary to underlying volvulus formation. By better understanding the complications induced by hypothyroidism, physicians may be able to prevent further life-threatening outcomes with early management and intervention.

## Introduction

It has been postulated that thyroid hormones play an undetermined role in the mediation of catecholamines that may result in gastrointestinal (GI) symptoms. Hypothyroidism can impair esophageal motility and gastric emptying resulting in constipation and abdominal distention. However, it has not been well documented in the literature that long standing untreated hypothyroidism can result in significant complications such as volvulus. Although chronic intestinal pseudo-obstruction in the bowel has been shown resulting in mega-small bowel or mega-duodenum due to digestive motility dysfunction, an underlying etiology of hypothyroidism may go overlooked as it may be concealed by concomitant illnesses or other potential sources. Volvulus which is a form of malrotation may involve a loop of the bowel resulting in twisting at a focal point along the mesentery attached to the intestinal tract. Properly managed hypothyroidism can reduce the risk of complications of volvulus including strangulation, perforation, fecal peritonitis, gangrene, and recurrent volvulus.

## Case Report

A 71-year-old female with a past medical history of hypertension, hypothyroidism, and hyperlipidemia presented to the emergency room with complaints of abdominal pain and distension with diarrhea for the past 2 days that has been getting progressively worse. The abdominal pain was located in the midepigastric region and radiated to the flanks. Soon after eating, she would have multiple episodes of diarrhea and thus her appetite over the past day had significantly deteriorated. She denied any hematochezia, melena, or vomiting.

On physical examination, she had a blood pressure of 150/90, heart rate of 65 beats/min, respiratory rate of 12/min, and a temperature of 98.5 °F. Significant findings on examination demonstrated a distended abdomen with hyperactive bowel sounds. There was centrally located epigastric tenderness to light and deep palpation without any signs of guarding.

**Figure 1:**
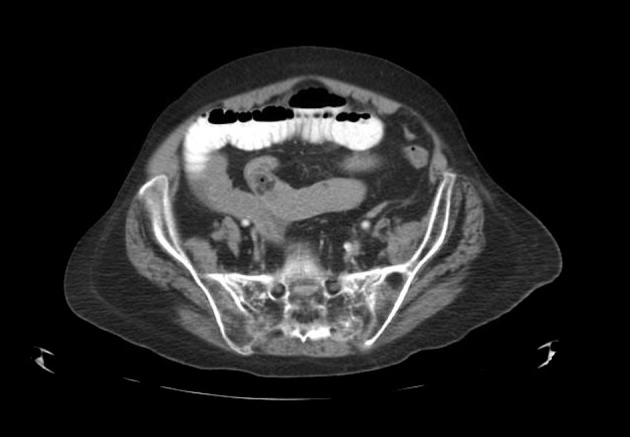
CT scan demonstrating moderate degree of diffuse small bowel dilation with air-fluid levels and in the distal small bowel a suspected intraluminal fatty mass visualized in the small bowel loop in the midpelvis.

Laboratory data showed leukocytosis of 13.4, hemoglobin of 13.6, hematocrit of 62, and platelets of 259. Sodium was 139, potassium of 4.2, chloride of 100, bicarbonate of 26, blood urea nitrogen of 30, creatinine of 1.2, and glucose of 111. Computed tomography of the abdomen showed small bowel dilation with air fluids suspected of small bowel obstruction. Intraluminal fatty mass was suggestive in the ileum and there was endometrial thickening with abnormal appearance of the uterus (Fig. 1).

The patient was admitted to the hospital and a surgery consult was placed. The patient was placed NPO, a nasogastric tube was placed, and normal saline was started at 100 mL/h. The patient was started on unasyn 1.5 g IV at 100 mg/h initially. The following day, the patient’s abdomen was found to be more distended. Her thyroid stimulating hormone (TSH) level returned measuring at 113 and her free T4 came back less than 0.023. The patient had been non-compliant and stated she had stopped taking her synthroid medications for several years. She was placed on synthroid, which would be advanced weakly.

The patient was taken for exploratory laparotomy where the small bowel was run from proximal dilated small bowel to an area where there was found to be an interval volvulus. In addition, there was evidence of hemorrhage within the mesentery at the point of volvulus as evidence of venous congestion. There was no evidence of an intraluminal process as was suggested by the CT scan. The obstruction was released with small bowel contents now dilating up the previously collapsed small bowel near the terminal ileum.

Her hospital course was further complicated by multiple episodes of coffee ground emesis with a drop in her hemoglobin. Endoscopy was suggestive of gastritis and after stabilization with advancement of her diet, she was discharged to subacute rehab after a few days.

## Discussion

Thyroid dysfunction impacts many organs in the body including the gut and viscera. When patients present with symptoms involving the digestive system, a thyroid etiology should be taken into consideration. Patients with significant hypothyroidism may even present with medical conditions and if not properly managed can lead to life-threatening complications. However, if an underlying etiology of hypothyroidism is not determined and managed, as was demonstrated in our patient, recurrence and future complication risk is high. GI motor dysfunction has been demonstrated as the cause of these symptoms but the underlying mechanism of action remains an enigma.

Hypothyroidism’s impact on the GI tract is likely multiple factorial with possible causes being attributed to neuromuscular dysfunction, myopathy, or alterations in hormone receptors. One of the most common symptoms is constipation as a result of peristalsis. However, our patient did not demonstrate these findings as she was having recurrent episodes of diarrhea. The constipation may be a result of hypothyroidism causing an inhibition of Cl^-^/HCO^3-^ anion exchange resulting in transepithelial flux transport [1]. Diarrhea, on the other hand, as seen in our patient, can be the result of increased bacterial growth which can itself be secondary to intestinal hypomotility [2-4].

Reports of motility dysfunction secondary to severe hypothyroidism have been reported but there remains a lack of data demonstrating significant symptoms suggestive of volvulus. Acute ileus has been shown to be a surgical emergency and is a rare complication of severe hypothyroidism. It has been postulated that it may be caused by myxedematous deposits causing a separation in the muscle fibers in the bowel wall from their ganglia [5]. Mild forms of ileus have been shown to be reversible with the use of thyroid hormone replacement but in complicated cases, it may be a surgical emergency as it can be life-threatening [6]. Some cases suggest that ileus may be a result of peripheral neuropathy affecting the gut. It has been suggested hypothyroidism can impact the peripheral nerves such as Schwann cell disease which can be a result of mucopolysaccharide deposition [6].

Volvulus, a condition caused by malrotation on its mesenteric axis, can result in obstruction of the bowel lumen which can impact the blood supply. Without surgery, a strangulated volvulus has a high mortality. It has been shown that chronic constipation is a known risk factor of volvulus and thus may be a secondary result of severe hypothyroidism. However, in our case report, the patient was having multiple episodes of diarrhea which suggests a potential alternate mechanism of action secondary to hypothyroidism.

Other than surgery, thyroid hormone replacement plays an important role in the management of ileus and volvulus in these patients. It is unclear whether thyroxine, triiodothyronine, or both are the best method of administration [7, 8]. Studies have shown that the route of administration of L-thyroxine however does not impact the outcome [9, 10]. Post-operatively, our patient was continued on thyroid hormone replacement with synthroid which was adjusted after weekly follow-up measurements as outpatient. Due to years of low thyroid function, there appears to be a correlation with progressive worsening of GI symptoms which may result in significant complications such as volvulus.

### Conclusion

Although surgically managed, volvulus has a risk of recurrence as an underlying condition such as hypothyroidism may be the culprit. Without treatment of hypothyroidism, there is a risk of future complications and recurrence that may result in perforation, gangrene, peritonitis, and other life-threatening conditions. Through this case discussion, physicians should be more aware and be able to recognize conditions which may be secondary to thyroid dysfunction.
